# Physiological and Morphological Responses of Hydroponically Grown Pear Rootstock Under Phosphorus Treatment

**DOI:** 10.3389/fpls.2021.696045

**Published:** 2021-11-11

**Authors:** Guodong Chen, Yang Li, Cong Jin, Jizhong Wang, Li Wang, Juyou Wu

**Affiliations:** ^1^College of Life Science and Food Engineering, Huaiyin Institute of Technology, Huai’an, China; ^2^College of Horticulture, Nanjing Agricultural University, Nanjing, China

**Keywords:** pear seedling rootstock, phosphorus, root system architecture, nutrients, photosynthesis

## Abstract

Phosphorus (P) is an essential macronutrient for the growth and development of fruit trees, playing an important role in photosynthesis, nucleic acid synthesis, and enzyme activity regulation. The plasticity of plant phenotypic has been investigated in diverse species under conditions of P-deficiency or P-excess. Based on these researches, P level fluctuations in different species result in different characteristics of the response. Nevertheless, little is known about the response of pear seedling rootstock (*Pyrus betulifolia* Bunge) to the changing of P levels. To explore the effects of different levels of P on the growth of pear seedling rootstock, we performed the hydroponic assays to determine and analyze the biological indexes including growth parameters, photosynthetic rate, root and shoot morphological traits, and concentrations of macro- and micronutrients. The results show that either deficiency or excess of P inhibited the growth and development of pear seedling rootstock. Root growth (down 44.8%), photosynthetic rate (down 59.8%), and acid phosphatase (ACP) activity (down 44.4%) were inhibited under the P-deficiency conditions (0mM), compared with normal P conditions (1mM). On the other hand, dark green leaves, suppression of root elongation (down 18.8%), and photosynthetic rate (down 25%) were observed under regimes of excessive P, compared with normal P conditions (1mM). Furthermore, the root concentration of not only P, but also those of other mineral nutrients were affected by either P treatment. In brief, these results indicated that a careful choice of P fertilizer supply is crucial to ensuring normal growth and development of pear seedling rootstock.

## Introduction

Phosphorus is one of the most important nutrients in the growth and development of fruit trees. It not only participates directly in the metabolism of sugar, protein, and fat, affecting the yield and quality of fruit trees (Carstensen et al., 2018; Qu et al., 2020; Siedliska et al., 2021), but it is also an indispensable component of the energy carrier ATP and the reducing agent NADP, generated by photosynthesis in plants (Shi et al., 2020; De Andrade et al., 2021), playing important roles in the process of photosynthesis, signal transduction, and physiological and biochemical regulation (Ham et al., 2018; Huang et al., 2019). Approximately 43% of the cultivated land in the world is deficient in phosphorus, with about two-thirds of the cultivated land in China being low in phosphorus (Wang et al., 2010). Therefore, with the rapid development of modern agriculture and increasing market demand, increasing the application of phosphorus fertilizer has become a quick way to improve soil fertility, and crop yield and quality. However, there are significant differences in the efficiency of phosphorus uptake and utilization among different crops, and different phenotypic symptoms are shown in response to different P levels, which can be reflected in the external morphology and internal physiological and biochemical processes of plants.

The use of rootstock in fruit production is inevitable, and rootstocks have a primary role in determining orchard efficiency, they are responsible for water and mineral uptake and provide anchorage for the tree (Guney, 2019; Cimen, 2020; Rufato et al., 2021). Commercial pear trees, such as Asian pear (*Pyrus pyrifolia* L.), are mainly propagated by grafting. The scion is grafted on rootstock, which determines most of the shoot traits, affecting the growth, yield, foliar disease resistance, and fruit characteristics of the grafted plants (Fisarakis et al., 2004), whereas the rootstock determines root traits such as pest and disease resistance, as well as overall pear tree size. However, as most of the cultivated land in China is lacking in phosphorus, the fruit growers tend to apply large amounts of phosphate fertilizer in an attempt to compensate, which will bring about losses to the production of certain crop plants (Veneklaas et al., 2012). Therefore, it is of vital significance to study the physiological phenotypic traits and the changes in mineral element concentrations in tissues of birch-leaf pear (*Pyrus betulifolia* Bunge), which is commonly used as the rootstock for commercial pear grafting, under phosphorus stress (deficiency or excess), in order to guide the balanced nutrient application to pear trees, to improve the yield and quality of pear, and to maintain soil fertility and protect the environment.

The utilization of soil phosphorus by fruit trees is achieved mainly through the active uptake process by roots along the inverse phosphate concentration gradient (Richardson et al., 2005). It is generally accepted that the utilization of phosphorus is realized by extruding H^+^ from the cell *via* the H^+^-ATPase ion pump, located on the vacuolar membrane, to generate a membrane potential as the driving force for phosphate uptake, with the aid of a protonated phosphate root carrier, which belongs to the co-transport mode of H^+^ and H_2_PO_4_^−^ (Yu et al., 2016). The epidermal cells of the root system of fruit trees are the main sites of phosphate accumulation in plants. The phosphate enters the xylem vessels through the symplast route and is then transported to the shoot of the plant for use by the fruit trees. Previous studies have shown that a series of morphological, physiological, and biochemical changes of plants under phosphorus stress, on the one hand, can reduce or increase the demand for phosphorus by reducing the growth rate, activating the use of phosphorus, or changing the process of carbon metabolism in plants (Brembu et al., 2017). On the other hand, the absorption of phosphate can be regulated by changing the root/shoot ratio, the morphology and configuration of root, or the level of organic acid anions, resulting from ACP activity, secreted by the roots (Tang et al., 2020). However, the morphological adaptation and physiological responses of different plants to phosphorus stress also show different characteristics. For example, in rubber tree (Moreira et al., 2013), *Stylosanthes guianensis* (Latief et al., 2020), soybean (Mo et al., 2019), and wheat (Shen et al., 2018), the growth indexes of total root length, root surface area, root volume, and root tip number were significantly reduced under low-phosphorus conditions, whereas the shoot resources were preferentially distributed to the roots, increasing the total surface area of the roots, the number of lateral roots, the root/shoot ratio, and the total root length under low-phosphorus conditions in cotton (Wang et al., 2018), tobacco (Zheng et al., 2013), and tomato (Marques, 2018). When *Stylosanthes* suffered from phosphorus stress, the concentrations of citric acid, succinic acid, and malic acid, and the activities of phosphatase, and the antioxidant enzymes superoxide dismutase, peroxidase, and catalase in the roots were significantly increased (Luo et al., 2020). In addition, the photosynthetic rate of leaf was significantly decreased, and the synthesis and transportation of soluble sugar were also affected by phosphorus stress in leaf, which lead to the shorter of plant height and less of leaf area (Veronica et al., 2017).

Excessive application of phosphate fertilizer will also cause a phosphorus stress, causing different degrees of damage to plants, leading not only to greater nutrient concentrations in the plant tissues, but also to decreasing soluble protein content and net photosynthetic rate in leaves, which, in turn, slows the growth and development of the plants. Moreover, phosphorus which has not been absorbed and utilized by plants leaches from the soil, causing environmental pollution and eutrophication of water bodies (Huang et al., 2012; Ye et al., 2015).

Identified as an advanced cultivation method in soilless culture, hydroponics directly contacts plant roots with nutrient solution to ensure plant growth and development. Compared with the traditional soil planting, hydroponics technology has the advantages of short growth cycle, effective resource saving, flexible cultivation condition, and reduced infection rate of diseases and pests (Shehzad et al., 2021). In addition, hydroponic method is capable of helping in the observation and management, as it can accurately control the growth and development process of plants (Sugiharta et al., 2019). Researchers have studied the phenotypic characteristics of plants through hydroponic method in the cultivation processes of diverse fruit species. For example, the hydroponic system was applied to investigate the reaction mechanism responding to Fe shortage in two grape rootstocks, simultaneously compare changes in morphological, physiological, and biochemical parameters (Marastoni et al., 2020). In apple, Qi et al. (2019) employed the hydroponic assay to explore the *Malus hupehensis* K^+^ uptake mechanism under drought stress. In citrus, Zhou et al. (2014) evaluated the effects of B-deficiency on plant growth and root-morphology traits *via* hydroponic assays in seven citrus rootstocks. Furthermore, some researchers utilized the hydroponics system to study the effects of mineral elements on plant phenotype in *Arabidopsis*, maize, and sunflower, respectively (Rivelli et al., 2010; Gruber et al., 2013; Kong et al., 2020).

Pear is one of the most important fruits, greatly appreciated by consumers around the world, because it is a flavorful and nutrient-rich fruit with medicinal effects. However, symptoms of phosphorus deficiency in pear trees and subsequent excessive applications of phosphate fertilizer reduce the growth, development, yield, and fruit quality of pear trees. Therefore, it is of great importance to study the response characteristics of pear trees to phosphorus stress to achieve the sustainable development of a modern fruit industry. In this study, the plant growth, root morphology, and changes in phenotypic symptoms of pear seedling rootstock (*P. betulifolia*) were analyzed by hydroponic system under both high- and low-phosphate stress, as the roots (contributed by the rootstock) are the principal initial site of phosphate stress. Furthermore, the effects on mineral element concentrations and photosynthetic rate were also determined under conditions of phosphorus deficiency or excess, to provide a theoretical basis for the balanced fertilizer application of phosphorus, to regulate root growth in pear.

## Materials and Methods

### Plant Materials and Treatment

In this study, seedlings of the birch-leaf pear (*P. betulifolia*) were used. Before the hydroponic experiments were set up, pear seeds were initially surface sterilized for 15min with 3% (v/v) sodium hypochlorite solution, then rinsed thoroughly with distilled water. The sterilized pear seeds were then soaked in distilled water for 24h before being placed in a box of expanded polystyrene foam filled with clean moist sand (5–10% moisture content), and were incubated at 4°C for 40days to achieve stratification. The stratified seeds were transferred into a growth chamber for 2days until the seeds germinated; the germinated seeds were then transplanted into 5-×10-hole black plastic trays of modules filled with vermiculite. After 14days, uniform seedlings were transplanted to half-strength nutrient solution containing 0.5mM KH_2_PO_4_, 1mM MgSO_4_·7H_2_O, 2mM Ca(NO_3_)_2_·4H_2_O, 2.5mM KNO_3_, 0.5mM NH_4_NO_3_, 0.83mg/L KI, 6.2mg/L H_3_BO_3_, 8.6mg/L ZnSO_4_, 0.25mg/L Na_2_MoO_4_, 0.025mg/L CuSO_4_, 0.025mg/L CoCl_2_, 22.3mg/L MnSO_4_, and 0.05mM Na_2_EDTA-Fe for a 1-week pre-culture period. Subsequently, the pear seedlings were placed into full-strength nutrient solution containing different concentrations of KH_2_PO_4_ (0, 0.5, 1, and 5mM), with the potassium concentration being balanced by the addition of 5, 4.5, 4, and 0mM KCl, respectively. The pH value of each nutrient solution was adjusted to 5.8 with 100mM KOH. The nutrient solution was replaced twice each week and ventilated for 30min every 3h by the combined action of a timer (Pinyi AL-06; China) and a ventilation pump (SUNSUN ACO, China). All the seedlings were cultured in a growth chamber (Jiangnan Instrument, Ningbo, China) with a light intensity of 800mmolm^−2^ s^−1^ of photosynthetically active radiation, and a light/dark regime of 14/10h at 28/22°C and 75% relative humidity. The pear seedlings were cultured for 5weeks, by which time the typical outward symptoms of P excess or deficiency had become apparent.

### Harvest and Morphological Parameter Measurement of Pear Seedlings

After 5weeks of treatment, the pear seedlings were harvested and divided into leaf, stem, and root tissues. The fresh weight (FW) of root, stem, and leaf were measured on an electronic analytical balance (FA, 2014), then the root/shoot ratio was calculated. The leaf area of each seedling was measured using a leaf area meter (Li-3100C; LI-COR Biosciences Inc., Lincoln, NE, United States). The length of the main root and the plant height were measured using a scaled ruler. Representative leaves and roots from seedlings from the different P treatments were imaged by a PowerShot Pro 1 camera (Canon, Tokyo, Japan).

### Measurement of Root System Architecture of Pear Seedlings

Pear seedlings were harvested at random from the various P-treatment groups and the root systems were rinsed with distilled water before assessment of root-related parameters. Total root length, total root number, total root volume, total root surface area, and the mean root diameter were determined for each plant, using an Epson digital scanner (Expression 10000XL 1.0; Epson Inc., Japan), and the root images were analyzed with WinRhizo software (Regent Instruments Canada Inc., 2013).

### Measurement of Photosynthetic Parameters of Pear Seedlings

Photosynthetic parameters measurement was assessed according to Wang et al. (2018). The brief process is as follows: Net photosynthetic rate (*P*_n_), intercellular CO_2_ concentration (*C*_i_), transpiration rate (*T*_r_), and stomatal conductance value (*G*_s_) were measured at different P treatments using a portable photosynthesis measuring system (LI-COR 6400, Lincoln, NE, United States). These measurements were performed on sunny days between 9:00 and 11:30a.m. in each unit under the following parameters: the ambient CO_2_ concentration was 378μmolmol^−1^, light intensity of 2,000μmol(photon) m^−2^ s^−1^, and leaf air vapor pressure was 2.5±0.3kPa.

### Leaf Chlorophyll Concentrations of Pear Seedlings

Chlorophyll was extracted from 100mg fresh leaves taken from seedlings subject to various P treatments, by grinding the leaves with a mortar and pestle for 5min in 10ml of 85% (*v*/*v*) acetone. The homogenate was sieved through filter paper, and the filtrate was transferred into a 15-ml Falcon tube and adjusted to a set fixed volume using 85% (*v*/*v*) acetone. Absorbance of the sample extracts was determined at both 663 and 644nm with a UV-1800 spectrophotometer (AuCy, China). The concentration of chlorophyll *a* and *b* were calculated by the following equations: chlorophyll *a*=1.07×(A_663_)−0.094×(A_644_); chlorophyll *b*=1.77×(A_644_)−0.280×(A_663_); total chlorophyll=chlorophyll *a*+chlorophyll *b*. The unit for chlorophyll concentration was mgg^−1^ FW sample.

### Measurement of Mineral Element Concentrations in Pear Seedlings

The mineral element concentrations of pear seedlings were measured as described previously (Chen et al., 2018). For total N concentration, approximately 1mg dry weight of frozen root tissue from each P treatment was ground in a ball mill and used for analysis by a Kjeldahl apparatus (JK9870). For the determination of concentrations of other mineral elements, a known weight (0.1–0.9g dry weight) of roots from each replicate of each P treatment was put into a PTFE digestion tube and digested with nitric acid in a microwave digester (Ultraclave 4; MLS). The concentration of mineral elements was analyzed by ICP–OES (inductively coupled plasma–optical emission spectrometry; 6500 dual ICP–OES spectrometer; Thermo Fisher Scientific, Waltham, MA, United States).

### Assay of Acid Phosphatase Activity in Pear Seedlings

The assay of ACP (acid phosphatase) activity used was a slightly modified version of McLachlan’s method (McLachlan et al., 1987). In brief, roots and leaves harvested from seedlings exposed to the different P treatments were rinsed with distilled water, before being blotted carefully with tissue paper. Samples (1g) of root or leaf were snap frozen with liquid nitrogen and then ground in a mortar and pestle, before the ground sample was suspended in 10ml of precooled extraction buffer. After incubation in an ice-bath for 1h, the solution was centrifuged at 4°C and 10,000×*g* for 25min. An aliquot (1ml) of the supernatant was treated with 1ml buffer and 0.1ml 4-nitrophenyl phosphate, disodium salt (pNPP-Na2), then incubated for 10min at 30°C. After 10min, the reaction was stopped by adding 1ml 0.5M NaOH. ACP activity was then measured at 400nm, using a UV-1800 spectrophotometer (AuCy, China). The level of ACP activity, in terms of the amount of *p*-nitrophenol produced (nmol per FWmg^−1^ root or leaf per min), was calculated by the following equation: ACP activity (nmol/minmg)=[(*A*/0.019)×3.1·(*V*/*V*1)]/[10×*W*(g)], where *A*=absorbance value of the sample; *V*=total volume of the enzyme extract; *V*1=the measurement of volume of the enzyme extract; *W*=weight of sample; and 10=incubation time (min).

### Experimental Design and Statistical Analyses

The experiment was established in a completely randomized 1×4 factorial design, with one pear rootstock source being subjected to four P treatments. All data on physiological and biochemical parameters are represented as mean±standard deviation (*n*=3 biological replicates). Statistical analyses of the data were performed by analysis of variance (ANOVA), using the SPSS statistical package (IBM, Armonk, NY, United States). Differences between samples were statistically compared for significance using the Duncan’s multiple range test. A probability level of 0.05 was considered to be statistically significant.

## Results

### Effect of Different P Levels on Phenotypic Symptoms and Root Growth

**Figure 1:**
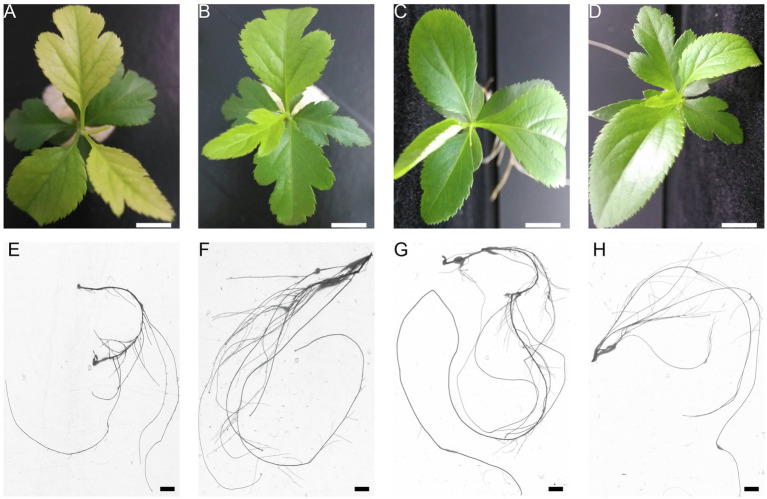
The effect of P treatments on phenotypic symptoms and root growth. Leaf color and expansion rate **(A–D)**, and root growth **(E–H)** of *Pyrus betulifolia* seedlings exposed to 0.0 **(A,E)**, 0.5 **(B,F)**, 1.0 **(C,G)**, or 5mM P **(D,H)**. The bars in **(A–H)** are 1cm.

To investigate the dynamic responses of pear seedlings to P stress (deficiency or excess) conditions, the phenotypic symptoms and root growth of pear seedlings grown under different concentrations of P were recorded using a photo-imaging system. Marked variations were observed in response to different P treatments (Figure 1). For example, the color of the pear seedling leaves changed from green to yellow or purple as the P supply level decreased (from 1 to 0mM), whereas the color of leaves showed symptoms of pale green under excessive P supply conditions, with the effect on leaf color being greater under P-deficient than under excess P conditions. In addition, leaf expansion rate was also significantly inhibited under P-deficient conditions. Conversely, there was no significant effect on leaf expansion rate under P-excess conditions. On the other hand, root growth was inhibited under either P stress (0 or 5mM), especially with respect to P deficiency (Figure 1). In brief, P-deficient or P-excess supply leads to an inhibition of pear seedling growth and development.

### Root Architecture Plasticity in Response to P Treatments

Root architecture refers to the structure and spatial distribution of the root system, which is mainly a three-dimensional spatial structure, formed to maximize the capture of water and nutrients in the soil. However, changes in nutrient availability and concentrations will affect the root architecture plasticity of fruit trees. Therefore, in order to explore the effects of different concentrations of P on the root architecture of pear seedling rootstock, the responses of the parameters total root length, total surface area, total root volume, mean root diameter, and root tip number to different P levels were analyzed. The total root length, surface area, volume, and root number on pear seedling rootstock changed significantly under P stress (deficiency or excess) conditions, but the root diameter was not significantly affected (Table 1). For example, over a range of deficient to adequate P conditions (0–1mM), the total root length, surface area, volume, and number of root tips of the seedlings increased with the increasing P supply concentration. The total length, surface area, volume, and number of root tips of the seedlings increased by 81.3, 72.6, 83.3, and 120.6%, respectively, when the seedlings were grown under the 1mM P level, compared with the P-deficient conditions (0mM). The total root surface area and root volume did not change significantly when the culture solution P concentration increased from the low-P concentration 0.5mM to the normal P concentration of 1mM, whereas the total root length and root tip number increased significantly by 9.5 and 11.5%, respectively, over the same concentration range. Excessive P supply (5mM) did not promote the root growth of pear seedling rootstock, but the total root length, root surface area, root volume, and root tip number were inhibited, resulting in decreases by 18.8, 15.7, 18.2, and 7.5%, respectively, compared to the corresponding values obtained at normal P levels (1mM).

**Table 1:** Effects of different P treatments on roots morphologic parameters of the pear seedling rootstock.

Treatments	Root length (cm)	Root surface area (cm^2^)	Root volume (cm^3^)	Root number	Diameter (mm)
0mM	48.76±2.56^d^	6.23±0.35^c^	0.06±0.01^c^	194.44+8.80^d^	0.41±0.02^a^
0.5mM	80.75±5.14^b^	10.11±0.74^ab^	0.10±0.01^ab^	384.67±16.84^c^	0.41±0.06^a^
1mM	88.4±4.90^a^	10.75±0.42^a^	0.11±0.01^a^	428.89±18.40^a^	0.39±0.07^a^
5mM	71.78±3.34^c^	9.06±0.32^b^	0.09±0.01^b^	396.89±14.61^b^	0.39±0.03^a^

Letters in superscript indicate whether mean values are significantly different between treatments (p < 0.05; different letters) or not (same letter).

### The Effect of P Treatments on Shoot Growth of Pear Seedlings

**Figure 2:**
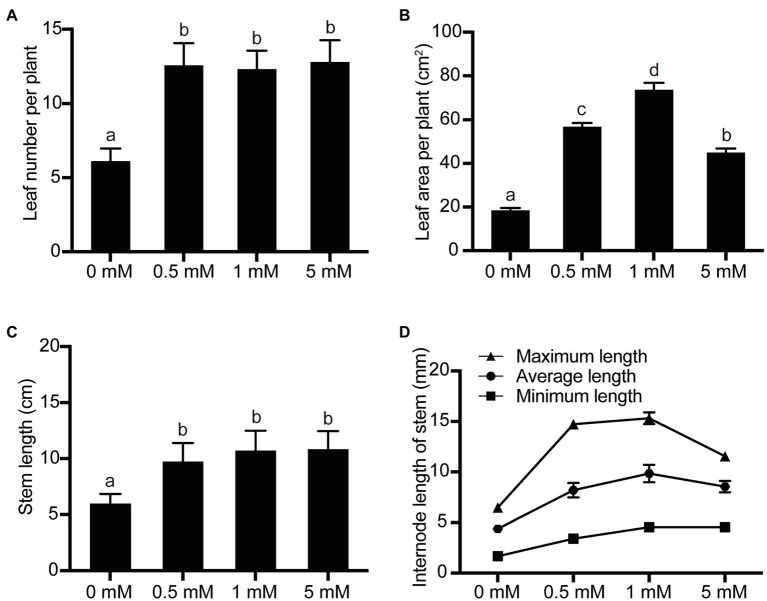
The effects of P treatments on pear seedling shoot growth. **(A)** The leaf number per pear seedling; **(B)** The leaf area per pear seedling; **(C)** The stem length per pear seedling; and **(D)** The internode length of stem per pear seedling. Data are presented as mean±standard deviation of three replicates, and each replicate is based on measurements made from three plants. Samples in a bar chart with a common superscript letter are not significantly different (*p*>0.05), according to the Duncan’s multiple range test.

The level of exogenous nutrient supply to fruit trees would also be expected to affect shoot growth in the pear seedlings. In order to study the shoot growth and development parameters of the seedlings under different P treatments, the parameters leaf number, total leaf area, plant height, and internode length were investigated. Over the concentration range 0 (deficient)–1 (normal mM P), the leaf number, total leaf area, plant height, and mean internode length of the pear seedlings all increased in response to increasing P supply concentration (Figure 2). For example, the number of leaves, total leaf area, plant height, and mean stem internode length increased by 100, 299, 78, and 123%, respectively, when the P supply level was 1mM, compared with the P-deficient level (0mM). On the other hand, the leaf number and plant height of the seedlings did not change significantly when the P supply level was increased from the low-P concentration 0.5 to 1mM, but the total leaf area and mean stem internode length changed significantly over the same range, increasing by 30 and 20%, respectively. In addition, the leaf number and plant height did not change significantly when P supply was raised from 1mM to 5mM, but both the total leaf area and the mean stem internode length were significantly inhibited at 5mM, decreasing by 39 and 13%, respectively, compared with the normal P (1mM) supply (Figure 2).

### The Effect of P Treatments on the Root/Shoot Fresh Weight Ratio

**Figure 3:**
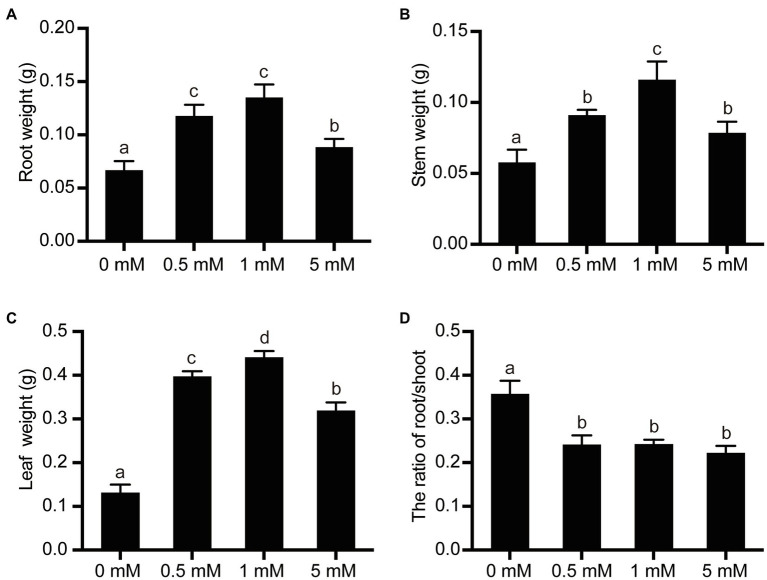
The effects of P treatments on the organ weight and root/shoot ratio in pear seedlings. **(A)** Root FW per pear seedling; **(B)** Stem FW per pear seedling; **(C)** Leaf FW per pear seedling; and **(D)** Root/shoot FW ratio. Data are presented as mean±standard deviation of three replicates, and each replicate is based on measurements made from three plants. Samples in a bar chart with a common superscript letter are not significantly different (*p*>0.05), according to the Duncan’s multiple range test.

To examine the effects of P stress (deficiency or excess) on the growth and development of the pear seedlings, the FW of leaves, stems, and roots of seedlings grown under different P treatments were measured and the root/shoot FW ratio was calculated. Because the number and total length of roots were both suppressed under low- or high-P stress conditions (Table 1), the root FW decreased concomitantly (Figure 3). Compared with seedlings grown under the normal P levels (1mM), seedlings grown under the low P (0mM) or excess P (5mM) treatments exhibited a 50% or 34% decrease in root FW, respectively (Figure 3). On the other hand, since the growth of the leaf and the stem were significantly inhibited under conditions of both P deficiency (0mM) and P excess (5mM), the leaf and stem FW initially increased with increasing P supply from 0 to 1mM and then decreased when the highest concentration of P (5mM) was supplied. The root/shoot FW ratio largely reflects the distribution of the photosynthetic product between the root and shoot. The experimental results showed that the ratio of root/shoot FW was significantly increased under conditions of P deficiency, due mainly to the fact that shoot FW appears to be more sensitive to low P conditions than does root FW. However, no significant differences in the root/shoot ratio were observed between the 1mM (normal) and 5mM (excess) levels of P supply, due mainly to the fact that both root and shoot growth were suppressed under the condition of excessive P supply, leading to similar changes in root and shoot FW.

### The Effect of P Treatments on Photosynthetic Parameters

**Figure 4:**
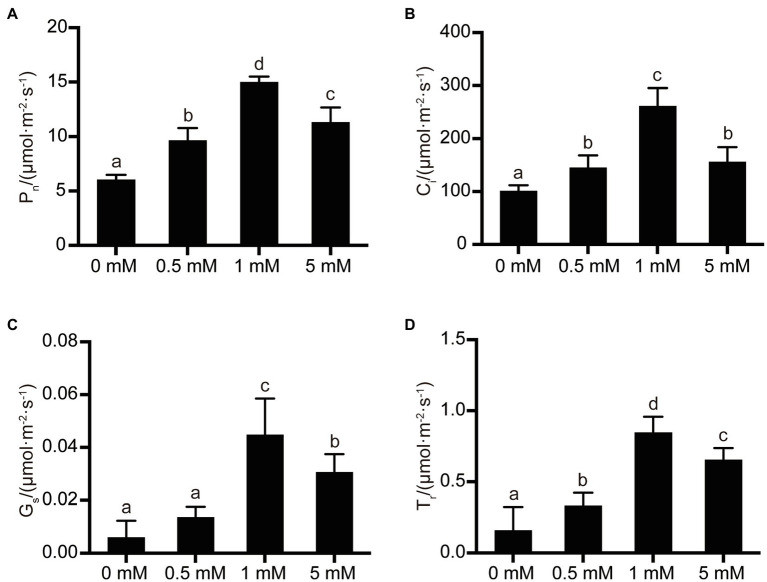
The effects of P treatments on photosynthetic parameters in pear seedling leaves. **(A)** The net photosynthetic rate of pear seedlings under different P levels; **(B)** The intercellular CO_2_ concentration of pear seedlings under different P levels; **(C)** The stomatal conductance of pear seedlings under different P levels; and **(D)** The transpiration rate of pear seedlings under different P levels. Data are presented as mean±standard deviation of three replicates, and each replicate is based on measurements made from three plants. Samples in a bar chart with a common superscript letter are not significantly different (*p*>0.05), according to the Duncan’s multiple range test.

The synthesis of carbohydrates by fruit trees is mainly achieved by leaf photosynthesis, with the important photosynthetic parameters being mainly the rate of net photosynthetic rate (*P*_n_), intercellular CO_2_ concentration (*C*_i_), transpiration rate (*T*_r_), and stomatal conductance value (*G*_s_). As shown in Figure 4, the photosynthetic parameters exhibited by pear seedling rootstock leaves were significantly affected by the different P levels supplied to the seedlings. Generally, the rate of net photosynthesis, intercellular CO_2_ concentration, stomatal conductance value, and transpiration rate of pear seedling rootstock leaves increased with increasing P supply level. The rate of net photosynthesis, intercellular CO_2_ concentration, stomatal conductance value, and transpiration rate increased by 149, 159, 650, and 433%, respectively, when P supply concentration was increased from the P-deficiency level (0mM) to the normal P concentration (1mM). In addition, the rate of net photosynthesis, intercellular CO_2_ concentration, stomatal conductance value, and transpiration rate increased by 58, 44, 127, and 109%, respectively, when the P level at which pear seedling rootstock was grown increased from P-deficiency levels (0mM) to low-P levels (0.5mM). However, it is worth noting that the net photosynthesis rate, intercellular CO_2_ concentration, stomatal conductance value, and transpiration rate of leaves were inhibited when the P supply level increased from normal P levels (1mM) to excessive P (5mM), decreasing by 25, 40, 32, and 23%, respectively.

### The Response to P Treatments of Chlorophyll Concentrations

**Figure 5:**
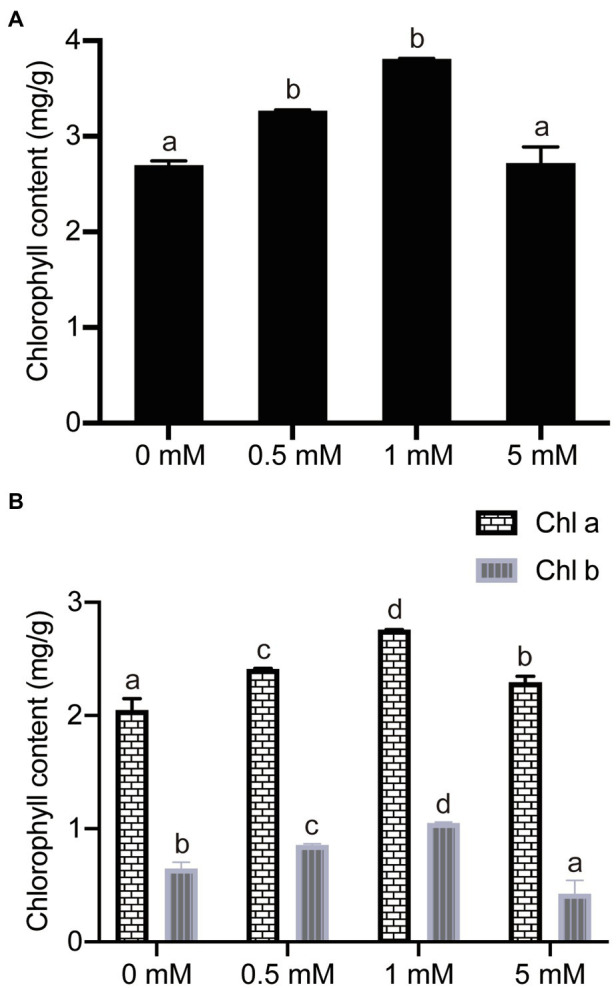
The effects of different P treatments on the chlorophyll concentrations in pear seedlings. **(A)** Total chlorophyll concentrations; and **(B)** Chlorophyll *a* and chlorophyll *b* concentrations. Data are presented as mean±standard deviation of three replicates, and each replicate is based on measurements made from three plants. Samples in a bar chart with a common superscript letter are not significantly different (*p*>0.05), according to the Duncan’s multiple range test.

In order to investigate the impact of P concentration on leaf chlorophyll concentrations, the leaf chlorophyll concentrations were analyzed in the pear seedlings from the four different P concentrations. Comparisons of the data showed that the total leaf chlorophyll concentration decreased significantly by 29 and 13% in seedlings grown under P-deficient (0mM) and low-P (0.5mM P) conditions, respectively, compared with seedlings supplied with a normal (1mM) P level (Figure 5A). Furthermore, the synthesis of total chlorophyll was also significantly suppressed under excess P (5mM) conditions, decreasing by 29% in the seedlings grown under 5mM P conditions, compared to those grown under a normal P supply (1mM, Figure 5A). On the other hand, the responses of either chlorophyll *a* or chlorophyll *b* concentrations to increasing P concentration in the hydroponic growth medium were similar to those of total chlorophyll concentration (Figure 5B). In addition, the concentration of chlorophyll *a* was more than three times that of chlorophyll *b* under the four P treatment conditions (Figure 5B). In brief, the experimental results showed that P stress (either deficiency or excess) inhibits the synthesis of chlorophyll, resulting in a decreased chlorophyll concentration, which may occur because P is an essential component in some photosynthetic enzymes.

### Effects of Different P Levels on Acid Phosphatase Activity in Pear Seedlings

**Figure 6:**
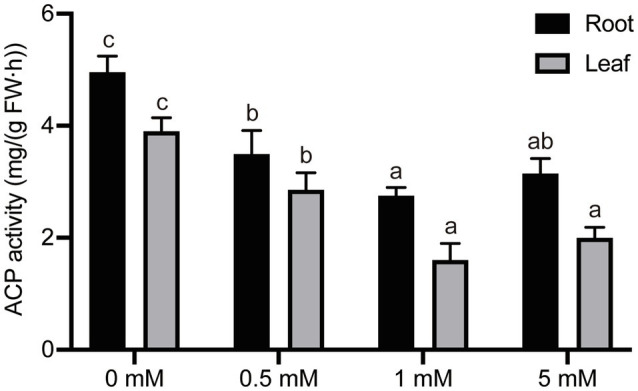
The effect of different P treatments on the acid phosphatase (ACP) activity in pear seedlings. Response of intercellular ACP activity in pear seedling root and leaf tissue to P concentration. Data are presented as mean±standard deviation of three replicates, and each replicate is based on measurements made from three plants. Samples in a bar chart with a common superscript letter are not significantly different (*p*>0.05), according to the Duncan’s multiple range test.

Intracellular ACP is a key enzyme in the release by hydrolysis and mobilization of phosphate in vacuoles into inorganic phosphorus. ACP is an inducible enzyme, the activity of which is affected by the availability of organic phosphorus, and it also plays an important role in the metabolism and reuse of organic phosphorus (Barker et al., 1974; McLachlan et al., 1987; Shen et al., 2018). Therefore, we researched the effects of seedling culture under different concentrations of exogenous P on ACP activity in the root and leaf tissue of pear seedlings. P-deficiency (0mM) or low-P treatments (0.5mM P) significantly induced ACP activity in pear roots by 80 and 27%, respectively, compared with seedlings cultured under normal (1mM) P supply (Figure 6). However, no significant difference in ACP activity was detected between seedling roots grown under conditions of normal or excess P concentration. Furthermore, the ACP activity in leaves was also significantly influenced by the different P treatments (Figure 6). The activity of ACP in leaves fell with an increase in P concentration within the range 0–1mM P. Seedlings grown under P-deficient or low-P (0 or 0.5mM, respectively) conditions resulted in a 144% or 78% increase in ACP activity, respectively, compared with activities in leaves of seedlings grown under the normal P levels (1mM).

### The Effect of P Treatments on Mineral Element Concentrations in Pear Seedling Roots

**Figure 7:**
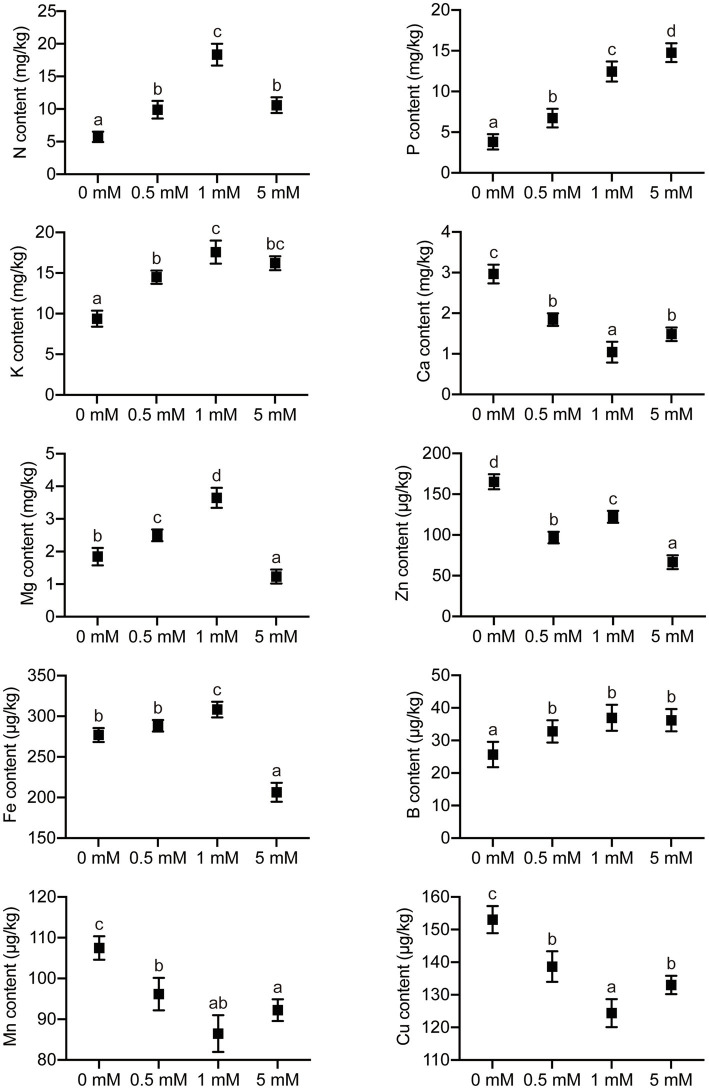
The effects of P treatments on the concentrations of elements in pear seedlings. Response of concentrations of elements in pear seedling roots to P supply concentration. Data are presented as mean±standard deviation of three replicates, and each replicate is based on measurements made from three plants. Samples in a bar chart with a common superscript letter are not significantly different (*p*>0.05), according to the Duncan’s multiple range test.

The concentrations of macronutrient elements (N, P, K, Ca, and Mg) and micronutrient elements (Fe, Mn, B, Zn, and Cu) were analyzed in the roots of pear seedlings, which had been grown under one of four levels of P. The P concentrations in the root gradually increased with increasing P supply when the level of P supply was between 0 and 1mM, but there was no significant increase when the P supply was in excess (5mM P; Figure 7). The concentrations of N, K, and Mg in the roots increased with increasing P level application over the range 0–1mM. However, the concentrations of N, K, and Mg in roots were significantly inhibited when the P supply was in excess (Figure 7). It is worth noting that the root concentration of Ca showed a response trend to the four levels of P different from those of N, K, and Mg, with a significant decrease in Ca concentration as the P level increased over the range 0–1mM. Not only the macronutrient concentrations but also the micronutrient concentrations were influenced by different P supply treatments. The concentrations of Mn and Cu displayed a trend similar to that of Ca in response to P supply concentration, decreasing markedly with increasing P supply (0–1mM), but the concentration of Mn and Cu increased significantly when the P supply was in excess (5mM; Figure 7). The concentration of Zn displayed a trend different from that of other micronutrient elements in response to increasing P levels, showing a significant decrease with increasing P supply (0–5mM). In addition, the root concentration of Fe was significantly inhibited only when P was supplied in excess, with no significant effects being observed at the other, lower P supply levels. Interestingly, the concentration of B did not change significantly under any P stress (deficiency or excess).

## Discussion

### Symptoms and Plant Growth

Previous studies have shown that plants often exhibit morphological and physiological changes under P-deficient conditions, such as stunted growth, reduction in leaf number, and decreased leaf area (Reddy et al., 2020). Studies in *Cucurbita pepo*, for example, demonstrated that the root/shoot ratio and the number of root tips were significantly increased under P-deficient conditions (Cao and Chen, 2012). In rice, plants exhibited stunted growth, with the foliage turning dark green color with reddish-purple tips and leaf margins under P-deficient conditions (Chen et al., 2014). In poplars, P deficiency caused chlorosis (pale yellow leaf color) and a decrease in leaf size after 60days of treatment (Zhang et al., 2014). Furthermore, an excessive P supply can also lead to a dark green leaf color, a dwarf habit, and delayed flowering in crop plants (Maharajan et al., 2018). In the present study, symptoms similar to those reported in the literature were observed in pear seedling rootstock grown under P-deficiency or excessive P supply conditions. P deficiency caused the development of purple or yellow color of the mature leaves. Taproot length, leaf expansion rate, and stem extension were all significantly inhibited under P-deficient conditions, but the root/shoot ratio showed a significant increase (relative to the value in seedlings at 1mM) under P-deficient conditions. However, excessive P treatment resulted in dark green leaves, with no significant change in leaf expansion rate, stem extension, or root/shoot ratio. The purple/yellow leaves under P-deficient conditions may be due to the accumulation of starch and anthocyanin in the leaves, leading to a decrease in nitrogen concentration in the leaves, resulting in the chlorotic symptoms of nitrogen deficiency. The increase in the root/shoot ratio may be due to inhibition of shoot growth being greater than that of the root growth under P-deficient conditions; when the P supply was excessive, the growth of the root system was inhibited as much as was that of the shoot system, so that the root/shoot ratio did not change. Thus, these results indicated that a moderate P fertilizer supply is crucial to ensuring normal growth and development in pear seedling rootstock.

### Effects of Phosphorus Deficiency on Root Architecture

Root architecture, the spatial distribution of a root system in the soil, has been shown to be important for plant P acquisition (Lynch and Beebe, 1995), and is highly plastic in its developmental response to P-stress conditions (Bates and Lynch, 1996; Hou et al., 2017). Specific impacts of P-deficiency on root growth have been identified in diverse species. For example, P deficiency significantly reduced the length of the principal root in *Lupinus albus* (Tang et al., 2013). In *Phaseolus vulgaris*, P deficiency reduced total root length by more than one-third, and the total lateral root length and the number of roots was also significantly reduced by P deficiency (Borch et al., 1999). In the grass *Neyraudia reynaudiana*, total root surface area, total root volume, and total root length were reduced in the P-poor compared with the P-rich treatments (Hou et al., 2017). In common bean, total root length and total root surface area were significantly reduced under the P-deficient conditions (Beebe et al., 2006), whereas, in *Arabidopsis*, primary root growth was suppressed under P-deficient concentrations (Williamson et al., 2001). Consistent with these published studies, the results of the current study showed that deficient or low levels of P (0 or 0.5mM) significantly decreased the root biomass, total root length, total root surface area, total root volume, and total root number in seedlings when compared with seedlings grown under normal P supply. However, response to exposure to excessive P resulted in a similar root architecture plasticity, with the total root length, total root surface area, total root volume, and root tip number decreasing by 18.8, 15.7, 18.2, and 7.5%, respectively, compared with the corresponding values from the seedlings grown under normal P levels (1mM). The ‘root architecture plasticity’ phenomenon is probably due to P stress affecting the carbon budget and the distribution of auxins and cytokinins. Furthermore, previous studies have reported that different plant species and populations show different responses to P deficiencies, such as in maize (Hajabbasi and Schumacher, 1994), *Fraxinus mandshurica* (Huang et al., 2019), and *Arabidopsis* (Bates and Lynch, 2000), where the values for total root length and total root surface area increased under P-deficient conditions, relative to normal P conditions, whereas the mean root diameter decreased. P deficiency caused a significant increase in primary root length, total root length, and the number of lateral roots after 8days of treatment in lentil (Sarker and Karmoker, 2009). Pandey reported a significantly greater total root surface area and total root volume in P-efficient mungbean genotypes grown under conditions of P stress (Pandey et al., 2014). P deficiency significantly promoted root hair density and the length of the basal and middle part of the primary root, as well as on the lateral roots, of the trifoliate orange, *Poncirus trifoliata* (Cao et al., 2013). Reasons for such findings may be that plants tend to allocate a greater proportion of biomass to the root system under P-deficient conditions (Hermans et al., 2006), with an increase in total root surface area, total root length, and root biomass, all traits positively associated with P uptake.

### Effects of P Levels on the Photosynthetic Characteristics of Pear Seedling Rootstock

P deficiency has been reported to lead to a decrease in chlorophyll concentration and photosynthetic rate (Zhang et al., 2014). Net photosynthetic rate is one of the most important indexes of plant physiology, reflecting the photosynthetic capacity of plants (Mukhopadyay et al., 2012; Zhang et al., 2014). Previous studies have shown that, with an increase in P supply, the net photosynthetic rate of *Camellia oleifera* seedlings showed a significant positive correlation with P concentration, increasing the accumulation of organic matter and promoting the growth of the plant (Fu et al., 2018). Similar results were obtained in the current study, with the net photosynthetic rate, intercellular CO_2_ concentration, stomatal conductance, and transpiration rate values of the seedlings being inhibited under P-deficient or low-P conditions. This showed that stomatal conductance of pear seedling rootstock was significantly correlated with transpiration rate, intercellular CO_2_ concentration, and net photosynthetic rate, reflecting the fact that stomata are the main channels for gas exchange between plant leaves and the outside world. The synthesis of chlorophyll was affected by inhibition of the uptake of nitrogen under the P-deficient or low-P conditions, which caused decreased leaf photosynthetic rate, delayed leaf growth, and reduced water requirements, as well as decreased transpiration rate, stomatal closure, and intercellular carbon dioxide concentration. Ultimately, net photosynthetic rate and plant growth decreased under P-deficient conditions. Furthermore, the synthesis of chlorophyll in leaves of pear seedling rootstock was inhibited under excess P conditions (5mM P), which affected the net photosynthetic rate, resulting in a decrease in transpiration rate, stomatal conductance, and intercellular CO_2_ concentration. These results are similar to those reported in the previous study by Boyce et al. (2006) on the Rocky Mountain bristlecone pine, *Pinus aristata*, where P had a marked influence on the photosynthetic rate of the plants. The results from the current study showed that, compared with the P-deficient treatments, the normal phosphorus supply (1mM P) increased the chlorophyll concentration in pear seedling rootstock, and subsequently enhanced the absorption of incident light energy, so that the net photosynthetic rate increased. This enhancement of photosynthesis was also probably the main cause of the significant increase in the biomass of pear seedling rootstock with the increase in P supply level.

### Effects of P Levels on Mineral Element Uptake by Pear Seedling Rootstock

Excessive or inadequate P supplies will affect the uptake of P by plants, a phenomenon which has been widely reported in rice, soybean, and other crops (Insalud et al., 2006; Vandamme et al., 2013; Pandey et al., 2014). Previous studies had shown that the P concentration in crop roots increases with increased P application level (Heuer et al., 2017). In the current study, the results showed that the P concentration in roots of pear seedling rootstock increased significantly with the increase in P level in the nutrient solution, indicating that the size of the root system of the pear seedlings was highly responsive to the exogenous P concentration. An appropriate amount of P supplied to the plants was conducive to the uptake of P by the roots, a finding which was consistent with results from tomato, rice, and poplar (Teng and Timmer, 1990; Wang et al., 2015; Wu et al., 2017). In addition, there were close correlations between concentrations of different plant mineral elements. Different levels of P supply not only affect the accumulation of P in plant roots, but also affect the uptake of other elements, such as nitrogen, potassium, calcium, magnesium, iron, copper, zinc, etc. (Su et al., 2015). The uptake of nitrogen, potassium, and magnesium by the root system of pear seedling rootstock was also inhibited under P-deficient or low-P conditions (0–0.5mM). The uptake of nitrogen, potassium, and magnesium into the roots generally increased with increasing P level; when the P supply level was excessive (5mM), not only was the accumulation of P in the roots reduced, but also the uptake of nitrogen, potassium, and magnesium in roots was inhibited. This result indicated that the uptake of N, K, and Mg by pear seedling rootstock operated synergistically with that of P. The low concentration of K and Mg in roots of pear seedling rootstock grown under low P conditions may be caused by the secretion of organic acids from the roots, accompanying the outflow of potassium and magnesium ions, while the low N concentration may be due to the loss of a large amount of potassium, resulting in the inhibition of N uptake (E et al., 2005; Xiao et al., 2014). Effects of P level on the concentration of calcium in roots are related to the species and populations of plants involved in the studies. The results from the current study showed that the calcium concentration in pear seedling roots grown under P-deficient or low-P conditions (0–0.5mM) decreased significantly with increasing P supply, indicating that the low-P environment is conducive to the uptake and utilization of calcium. When the P supply level is excessive (5mM), the concentration of calcium in roots increased, which indicated that the absorption of calcium by pear seedling roots was antagonistic to the supply level of P, a finding similar to that from a previous study on rapeseed (Hao et al., 2009). Furthermore, the results from the current study showed that concentrations of iron and boron did not change significantly with an increase in P supply level (0–1mM), while the concentrations of Fe and Zn in roots decreased significantly at the higher P level (5mM), indicating that excessive P levels inhibited the uptake of Fe and Zn by roots. The uptake of Mn, Cu, and Zn by roots was similar to that of Ca under the P-deficiency or low-P levels (0–0.5mM), gradually decreasing with increasing P supply over the range 0–1mM, indicating that the uptake of Mn, Cu, and Zn by roots of pear seedling rootstock was antagonistic to that of P, a finding similar to that of a previous study on castor bean (Huang et al., 2018). In addition, the uptake of Zn was inhibited and that of Mn and Cu was promoted by roots of seedlings grown under conditions of excess P (5mM).

## Data Availability Statement

The original contributions presented in the study are included in the article/supplementary material, further inquiries can be directed to the corresponding authors.

## Author Contributions

GC designed the research, performed the experiments, and analyzed the results. YL, CJ, JW, and LW participated in carrying out the experiments. JW managed the experiments and participated in revising the final manuscript. All authors contributed to the article and approved the submitted version.

## Funding

This work was supported by Jiangsu Agriculture Science and Technology Innovation Fund (CX(19)2028), the Natural Science Research Project in Colleges of Jiangsu Province of China (20KJB210007), National Natural Science Foundation of China (31801842), and Talent Introduction Research Project for Huaiyin Institute of Technology (Z301B19573).

## Conflict of Interest

The authors declare that the research was conducted in the absence of any commercial or financial relationships that could be construed as a potential conflict of interest.

## Publisher’s Note

All claims expressed in this article are solely those of the authors and do not necessarily represent those of their affiliated organizations, or those of the publisher, the editors and the reviewers. Any product that may be evaluated in this article, or claim that may be made by its manufacturer, is not guaranteed or endorsed by the publisher.

## Abbreviations

P: Phosphorus
ACP: Acid phosphatase
ICP: Inductively coupled plasma
FW: Fresh weight
*P*_n_: Net photosynthetic rate
*C*_i_: Intercellular CO_2_ concentration
*T*_r_: Transpiration rate
*G*_s_: Stomatal conductance value
